# Recanalization of Extracranial Internal Carotid Artery Occlusion after i.v. Thrombolysis for Acute Ischemic Stroke

**DOI:** 10.1371/journal.pone.0055318

**Published:** 2013-01-28

**Authors:** Raimund Pechlaner, Michael Knoflach, Benjamin Matosevic, Michael Ruecker, Christoph Schmidauer, Stefan Kiechl, Johann Willeit

**Affiliations:** Department of Neurology, Innsbruck Medical University, Innsbruck, Tyrol, Austria; University of Regensburg, Germany

## Abstract

**Background:**

Although extracranial internal carotid artery (e-ICA) occlusion is a common pathology in patients undergoing intravenous thrombolysis for treatment of acute ischemic stroke, no data on e-ICA recanalization rate or potential effects on outcome are yet available.

**Methods and Results:**

This study included 52 consecutive patients with e-ICA occlusion and ischemic stroke undergoing standard intravenous thrombolysis. The rate of e-ICA recanalization was 30.8% [95%CI, 18.2–43.3], documented at 3.5 [2.0–11.8] (median [IQR]) days after stroke, as compared to 8.6% [95%CI, 3.5–13.7] in a series of 116 consecutive patients with symptomatic e-ICA occlusion not undergoing thrombolysis (P<0.001 for difference). Functional outcome three months after stroke did not significantly differ for those with or without e-ICA recanalization following intravenous thrombolysis (modified Rankin scale ≤2: 31.3% vs. 22.2%, odds ratio 1.6 [95%CI, 0.4–5.9], P = 0.506). In patients with e-ICA occlusion of atherothrombotic origin, recanalization resulted in most instances in residual high-grade stenosis (13 of 14).

**Conclusions:**

Recanalization of e-ICA occlusion after stroke thrombolysis occurred in about one third of patients. Although e-ICA recanalization had no significant effect on patient outcome, control sonography in the early days after thrombolysis is recommended for the detection of potential residual e-ICA stenosis.

## Introduction

In acute stroke therapy, recanalization of the occluded intracranial vessels is decisive for a favourable clinical outcome, and the probability of vessel re-opening strongly depends on the segment affected [1]–[5]. Extracranial internal carotid artery (e-ICA) occlusion is a common finding in ischemic stroke [6] and is frequently suspected as source of embolism. No data are available on probability or prognostic significance of e-ICA recanalization following standard intravenous thrombolysis, although this information is of potential relevance to patient management.

## Methods

This retrospective analysis of prospectively collected data included 52 consecutive patients who underwent intravenous thrombolysis for treatment of acute ischemic stroke attributable to e-ICA occlusion. The patients were 65.2±17.4 (mean±SD) years old, 65.4% (n = 34) were male and all of Western European descent. All patients were treated at the stroke unit of Innsbruck University Hospital between January 1, 2002 and December 31, 2010 according to standard thrombolysis protocols as described previously [1], [7]. The decision in favour of or against thrombolysis was made on an individual basis using standard criteria (NINDS criteria). Patients older than 80 years were treated if otherwise healthy and eligible. Patients with pre-existent carotid occlusion ipsilateral to the acute stroke as well as one patient undergoing endovascular treatment in the acute phase of stroke were not considered. e-ICA occlusion was defined as occlusion of the ICA involving the cervical (C1 in Bouthillier nomenclature) segment.

Aetiology of ICA occlusion was classified as carotid dissection, cardiac embolism, or atherothrombosis. A putative cardio-embolic origin was assumed in the case of a hypoechoic occlusion without explicit detection of a plaque formation at the carotid bifurcation and presence of atrial fibrillation. Most of these occlusions spared the carotid bifurcation.

Vessel status was assessed by experienced stroke neurologists with utrasound using a 10-MHz linear transducer on a General Electrics Logiq 7 (Milwaukee, WI, USA). Initial clinical and ultrasound assessments were made on admission, before administration of intravenous thrombolysis. Vessel patency after thrombolysis was evaluated by high-resolution doppler assisted duplex imaging. A residual stenosis ≥70% according to ECST criteria was diagnosed if peak systolic velocity was >200 cm/s. Based on the earliest available control ultrasound examination, patients were classified in one of three categories (persistent occlusion, residual stenosis ≥70%, or recanalization without residual stenosis ≥70%, according to ECST criteria). The modified Rankin Scale (mRS) was used to gauge functional outcome three months after stroke. It was ascertained by telephone interview with the patient if feasible, by telephone interview with a caregiver otherwise.

Two additional patient series were recruited at the same centre. The first comprised 40 consecutive patients undergoing intravenous thrombolysis for treatment of ischemic stroke attributable to intracranial ICA occlusion (C2-C7 without involvement of C1). The second (n = 116) consisted of consecutive patients with acute stroke or transient ischemic attack attributable to e-ICA occlusion who did not receive intravenous thrombolysis. Characteristics of the three patient groups are shown in Table 1. The three patient groups were consecutive patient series including all patients presenting to Innsbruck University Hospital within the survey period.

**Table 1:** Baseline characteristics of acute ischemic stroke patients with internal carotid artery occlusion grouped according to occlusion localization and administration of systemic thrombolysis.

Localization	extracranial	intracranial	extracranial
Systemic thrombolysis	yes	yes	no
n	52	40	116
Affected side (right/left/both)	44.2/53.8/1.9	45.0/55.0/0.0	45.2/53.9/0.9
NIH-SS on admission	16.1±5.7	18.9±4.9	8.6±8.5
**Demographic variables**			
Age, years	65.2±17.4	69.2±12.6	67.1±12.6
Male sex	65.4	55.0	71.6
**Risk factors**			
Hypertension	61.5	67.5	74.8
Dyslipidemia	29.4	30.6	53.5
Smoking	26.9	12.5	38.2
Prior stroke or TIA	17.3	20.0	27.6
**Concomitant diseases**			
Atrial fibrillation	17.3	37.5	21.0
Diabetes mellitus	15.4	20.0	26.7
Coronary heart disease	12.0	22.2	23.5
**Prior Medication**			
Antiplatelet agents	26.9	27.5	44.4
Anticoagulants	0.0	4.8	8.1
Antihypertensive agents	48.0	51.4	60.6
Statins	10.0	2.7	14.1

Data are given as percentage, except NIH-SS on admission and age, which are given as mean ± standard deviation. “Prior Medication” refers to pharmacotherapy received by the patient up to the event. NIH-SS, National Institutes of Health Stroke Scale; TIA, transient ischemic attack.

Data were extracted from the local thrombolysis and ICA occlusion databases and the Austrian Stroke Unit Registry [1], [7]. The former two sources are part of the hospital standard quality control protocol, and as such require no explicit ethics committee approval or informed consent. The Austrian Stroke Unit Registry is part of a governmental quality assessment programme for stroke care in Austria financed by the Federal Ministry of Health under the Federal Law Promoting Quality in Health (Gesundheitsqualitätsgesetz). All data were anonymized. This research received no specific grant from any funding agency in the public, commercial or not-for-profit sectors.

Statistical analyses were performed using R, version 2.15.0. Differences in clinical outcome between patients with and without recanalization were tested using Fisher's exact test or the Cochran-Armitage trend test. Logistic regression was used to adjust for baseline National Institutes of Health Stroke Scale and age. All confidence intervals are reported at the 95% level and two-sided P-values <0.05 were considered significant.

## Results

**Figure 1:**
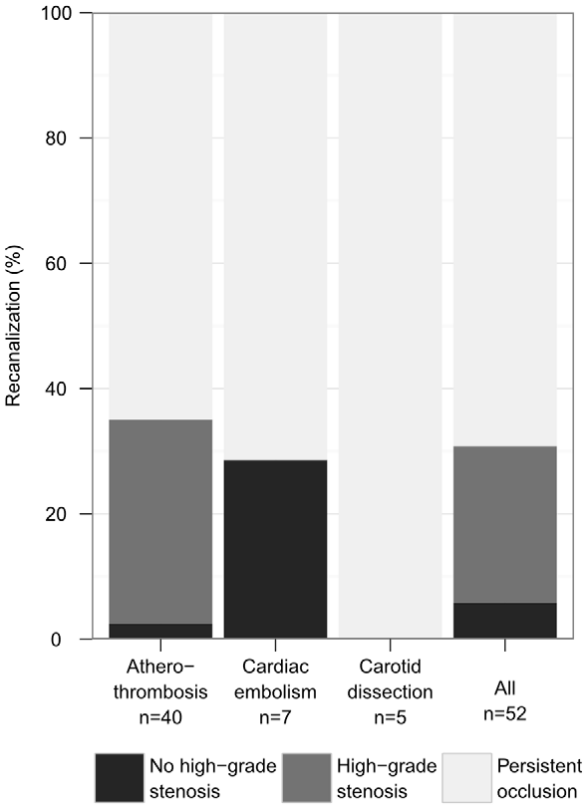
Recanalization rate for extracranial internal carotid artery occlusion after thrombolysis by aetiology. Aetiology was classified as carotid dissection, cardiac embolism, or atherothrombosis. The first control ultrasound exam was performed at a median 3.5 days after stroke.

**Figure 2:**
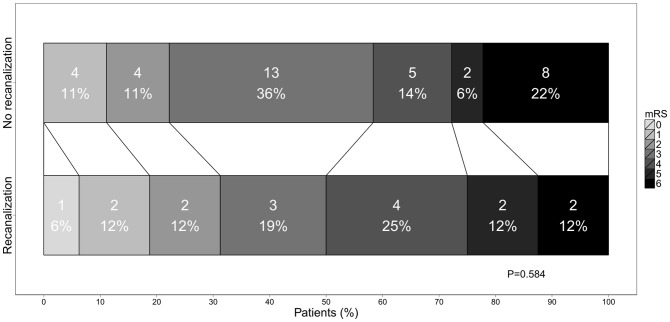
Three-month functional outcome in patients with vs. without recanalization of extracranial internal carotid artery occlusion after thrombolysis. Comparison of the modified Rankin Scale (mRS) distributions between the two groups. P by Cochran-Armitage trend test.

Baseline characteristics of the three groups are presented in Table 1. Patient characteristics of 52 patients with e-ICA occlusion who underwent thrombolysis were similar in those with and without recanalization. Median onset-to-treatment time was 122.5 minutes (IQR 99.3–150). Recanalization rate was 30.8% [95%CI, 18.2–43.3] (n = 16/52). Recanalization was documented at a median of 3.5 (IQR 2.0–11.8) days after stroke. In four patients the time interval between stroke onset and control sonography exceeded one week. At 35.0% [20.2–49.8] (n = 14/40) recanalization rate was highest in e-ICA occlusion due to atherothrombosis, and residual high-grade stenosis was observed in the majority of these patients (n = 13/14). In e-ICA occlusion due to cardiac embolism, 28.6% [0.0–62.0] (n = 2/7) showed recanalization, all without residual high-grade stenosis. No recanalization in the first control sonography was documented in e-ICA occlusion due to vessel dissection (n = 0/5) [Fig. 1]. Three months after stroke, functional outcome did not significantly differ for patients with or without e-ICA recanalization (mRS≤2: 31.3% vs. 22.2%, odds ratio (OR) 1.6 [0.4–5.9] P = 0.506; Table 2, for individual mRS categories see Fig. 2). Results were similar under adjustment for baseline NIH-SS and age (P = 0.262). When focusing on atherothrombotic e-ICA occlusion only (n = 40), functional outcome with respect to recanalization was as follows: mRS≤2: 35.7% vs. 19.2% in patients with persistent occlusion, OR 2.3 [0.5–10.1] P = 0.278.

**Table 2:** Outcome parameters of the three groups.

Localization	extracranial	intracranial	extracranial
Systemic thrombolysis	yes	yes	no
Recanalization rate	30.8	30.0	8.6
Time to recanalization, days	3.5 (9.8)	1.5 (5.0)	5.5 (3.8)
mRS at 3 months	3.5±1.7	4.0±2.0	2.5±1.9
mRS ≤2 overall	25.0	20.0	52.2
mRS ≤2 with recanalization	31.2	50.0	44.4
mRS ≤2 without recanalization	22.2	7.1	53.1

Data are given as percentage, except mRS at 3 months, which is given as mean ± standard deviation, and time to recanalization, which is given as median (interquartile range). Presence of recanalization was judged based on the first control ultrasound, which was performed at a median 3.5 days after stroke. mRS, modified Rankin Scale.

In the first comparator group of 40 patients with intracranial ICA occlusion who received thrombolysis, recanalization occurred in 30.0% [15.8–44.2] (n = 12/40). This is almost identical to the rate found in this study for extracranial and in others for intracranial ICA occlusion [8]. Recanalization was documented at a median of 1.5 (IQR 1.0–6.0) days after stroke and was crucially associated with good functional three-month outcome (mRS≤2: 50.0% vs. 7.1% with persistent occlusion, OR 13.0 [2.1–81.0], P = 0.0048; Table 2).

In the second comparator group of 116 patients with e-ICA occlusion who did not receive intravenous thrombolysis, the rate of spontaneous recanalization was 8.6% [3.5–13.7] (n = 10/116), documented at a median of 5.5 (IQR 3.3–7.0) days after stroke/TIA, and recanalization was not associated with three-month functional outcome (mRS≤2: 44.4% vs. 53.1% in patients with persistent occlusion, OR 0.7 [0.1–3.6], P = 0.732; Table 2). Accordingly, the e-ICA recanalization rate was significantly higher in patients with intravenous thrombolysis (OR 4.7 [1.8–12.6], P<0.001).

## Discussion

Although e-ICA occlusion is a common pathology in stroke patients undergoing intravenous thrombolysis, the frequency of e-ICA recanalization and potential implications for patient management and outcome are hitherto unknown. This contrasts with intracranial vessel occlusion, for which substantial knowledge about recanalization probabilities following acute stroke therapy has accumulated over recent years.

Our study indicates that the extracranial carotid artery reopens in about one third of stroke patients treated with standard intravenous thrombolysis (Fig. 1), which is more than three times the rate of e-ICA recanalization in stroke patients not undergoing thrombolytic therapy. The only previous report on this issue derives from an angiography-based rtPA dose-finding study conducted in the late 80 s. In that evaluation, two of 23 e-ICA occlusions (8.7%) recanalized within 24 hours after administration of various doses of duteplase [5].

Unlike for intracranial vessels, recanalization of e-ICA occlusion had little if any effect on patient outcome (Fig. 2), but for the treating stroke physician may pose the challenge of a residual high-grade stenosis. Indeed, in most patients with atherothrombotic e-ICA occlusion in our series a significant stenosis persisted (Fig. 1), and four (30.8%) were considered to benefit from surgical repair and subjected to carotid endarterectomy. Reasons for excluding patients from surgery included persistent severe disability (n = 3), early re-occlusion (n = 3), high age (n = 2), and spontaneous remission of stenosis (n = 1).

Therapeutical procedures alternative to intravenous thrombolysis, like stenting and mechanical thrombectomy, are feasible in patients with acute stroke and e-ICA occlusion [9], but have not yet been tested in randomized controlled trials and are thus not ready for broad clinical application. A recent small study (n = 21) [10] observed a favourable outcome in 7 of 13 patients with stroke and ICA occlusion undergoing i.v. thrombolysis, but only in 1 of 8 undergoing primary endovascular treatment, while a meta-analysis including 531 patients with ICA occlusion [11] reported a higher proportion of favourable outcome in the endovascular group (43.5 vs. 26.3%, P<0.0001).

This study has limitations including its size, and therefore, negative findings like the lack of significant association between e-ICA recanalization and outcome should be interpreted with caution. If the albeit small difference in functional outcome between subjects with and without e-ICA recanalization is assumed to be a true finding, post hoc power analysis indicates that a sample size 17 times larger would be needed for statistical significance (α = 0.05) to be achieved with a power of 0.8. Moreover, we did not conduct repeated control ultrasound examinations at standardized intervals after thrombolysis. We thus were not able to define the time at which e-ICA recanalization occurred and may have missed cases of temporary recanalization. Finally, ascertainment of vessel status relied on high-resolution Duplex sonography, with a reported sensitivity and specificity of 96% and 100%, respectively, for detection of e-ICA occlusion [12].

A key clinical implication of our data is the recommendation that an early control carotid sonogram be performed in stroke patients with e-ICA occlusion undergoing intravenous thrombolysis. In the case of recanalization and a persistent high-grade stenosis the necessity of surgical intervention has to be tailored individually by considering age, stroke severity and comorbidities. The timing of a potential intervention depends primarily on the infarct size and the presence or absence of hemorrhagic transformation and breakdown of the blood-brain barrier.
